# Supplementary material for: Mitigating gut microbial degradation of levodopa and enhancing brain dopamine: Implications in Parkinson’s disease
Source: Commun Biol. 2024 May 30;7:668. doi: 10.1038/s42003-024-06330-2 (PMC11139878; doi:10.1038/s42003-024-06330-2)
Supplement: Supplementary file 2 — Supplementary Information [file 42003_2024_6330_MOESM2_ESM.pdf]

SUPPLEMENTARY INFORMATION

Mitigating gut microbial degradation of levodopa and enhancing brain dopamine: Implications in Parkinson's disease

Gang Cheng,[1] Micael Hardy,[2] Cecilia J. Hillard,[3] Jimmy B. Feix,[1] Balaraman Kalyanaraman[1,*]

[1]Department of Biophysics, Medical College of Wisconsin, 8701 Watertown Plank Road, Milwaukee, WI 53226, United States
[2]Aix-Marseille Univ, CNRS, ICR, UMR 7273, Marseille 13013, France
[3]Department of Pharmacology and Toxicology and Neuroscience Research Center, Medical College of Wisconsin, 8701 Watertown Plank Road, Milwaukee, WI 53226, United States

*Corresponding author: Balaraman Kalyanaraman, Department of Biophysics, Medical College of Wisconsin, 8701 Watertown Plank Road, Milwaukee, WI 53226, United States; balarama@mcw.edu; 414-955-4000

Synthesis of Mito-ortho-HNK and Mito-PEG$_4$-HNK

Synthesis of mitochondria-targeted *ortho*-honokiol (Mito-*ortho*-HNK, Supplementary Figure 1) and the PEGylated mitochondria-targeted analog of honokiol (Mito-PEG$_4$-HNK, Supplementary Figure 2) are presented in the following sections. Mito-ATO, Mito-PEG$_2$-ATO, and Mito-PEG$_5$-ATO were prepared as previously described [1,2]. Their nuclear magnetic resonance (NMR) data are presented in Supplementary Figure 3 (presented toward the end of this file).

All chemicals and organic solvents were commercially available and were used as supplied. The reactions were monitored by thin layer chromatography using silica gel Merck ^{60}F254. Crude materials were purified by flash chromatography on Merck Silica gel 60 (0.040–0.063 mm). ^{1}H NMR spectra were recorded at 400.13 MHz respectively using a Bruker DPX AVANCE 400 spectrometer equipped with a quattro nucleus probe. ^{1}H NMR and ^{31}P were taken in deuterated chloroform ($CDCl_3$) using $CDCl_3$ and tetramethylsilane as internal reference respectively. Chemical shifts (δ) are reported in ppm and *J* values in Hertz.

Preparation of Mito-*ortho*-HNK ([10-[3,5'-diallyl-2'-hydroxy-(1,1'-biphenyl)-4-yl]-oxy]-decyltriphenylphosphonium bromide)

Mito-*ortho*-HNK was prepared by reacting 10-bromodecyltriphenylphosphonium bromide (Mito-Br) with honokiol (HNK) in the presence of potassium carbonate in dimethylformamide (DMF) (Supplementary Figure 1).

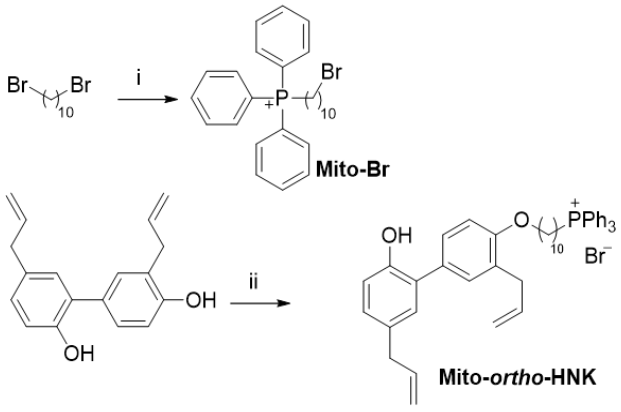

Supplementary Figure 1. Synthesis of Mito-*ortho*-HNK. Reagents and conditions: i, triphenylphosphine, neat, 90°C, 47%; ii, Mito-Br, K_2CO_3, DMF, 40°C, 38 h., 14%.

10-Bromodecyltriphenylphosphonium bromide. The procedure was adapted from Pan *et al.* [3]. A mixture of triphenylphosphonium (1 g, 3.8 mmol) and dibromide (5.7 g, 19 mmol) was heated at 90°C for 6 h. After cooling, the crude product was washed by ether (Et_2O) and purified by flash chromatography (pentane, Et_2O, and dichloromethane [CH_2Cl_2]/ethanol (EtOH) 9:1) to afford the corresponding phosphonium salt as a white solid (1 g, 47% yield).

^{31}P (400.13 MHz, $CDCl_3$) δ 24.32. ^{1}H NMR (400.13 MHz, $CDCl_3$) δ 7.85-7.65 (15H, m), 3.73-3.66 (2H, m), 3.40-3.34 (2H, m), 1.80-1.75 (4H, m), 1.31-1.20 (12H, m).

Mito-*ortho*-HNK. To a mixture of HNK (1.3 g, 4.9 mmol), anhydrous potassium carbonate (0.69 g, 4.9 mmol) in DMF (40 mL) was added 10-bromodecyltriphenylphosphonium bromide (2.8 g, 4.9 mmol). The mixture was stirred at 40°C for 24 h. The solvent was removed under vacuum, and the residue was taken up into water and extracted with CH_2Cl_2. The organic layer was dried over sodium sulfate, and the solvent was removed under reduced pressure. Purification by flash

chromatography (Et_2O, CH_2Cl_2, and CH_2Cl_2/EtOH) delivered the corresponding Mito-*ortho*-HNK (0.51 g, 14% yield).

^{31}P (400.13 MHz, $CDCl_3$) δ 24.58. ^{1}H NMR (400.13 MHz, $CDCl_3$) δ 7.86-7.65 (15H, m), 7.35-7.21 (2H, m), 7.04-6.89 (4H, m), 6.04-5.92 (2H, m), 5.57 (1H, s), 5.13-4.98 (4H, m), 3.99 (2H, t, *J* = 6.4), 3.87-3.75 (2H, m), 3.45-3.28 (4H, m), 1.84-1.74 (2H, m), 1.62-1.56 (3H, m), 1.51-1.12 (11H, m). ^{13}C NMR (75 MHz, $CDCl_3$) δ 156.3, 151.1, 137.9, 136.7, 134.9, 134.8, 133.7, 133.6, 131.8, 130.4, 130.3, 130.1, 129.6, 129.0, 128.6, 127.9, 127.8, 118.8, 118.2, 115.7, 115.6, 115.4, 111.7, 68.0, 39.4, 34.5, 30.4, 30.3, 29.3, 29.2, 29.19, 29.14, 29.08, 29.04, 25.9, 22.7, (d, *J* = 49.2), 22.6, (d, *J* = 4.2). HRMS calculated for Mito-*ortho*-HNK $C_{46}H_{52}O_2P$ $[MH]^+$ 667.3699, found, 667.3699.

Two-dimensional NMR was performed on a 600 MHz NMR equipped with TCI (triple resonance inverse) probe in manual mode. ^{1}H-^{1}H COSY (correlation spectroscopy), HSQC (heteronuclear single quantum correlation), HMBC (heteronuclear multiple bond correlation), and NOESY (nuclear overhauser effect spectroscopy) were used.

Preparation of Mito-PEG$_4$-HNK (2-(2-(2-(2-((3',5-diallyl-4'-hydroxy-[1,1'-biphenyl]-2-yl)oxy)ethoxy)ethoxy)ethoxy)ethyl)triphenylphosphonium bromide and (2-(2-(2-(2-((3,5'-diallyl-2'-hydroxy-[1,1'-biphenyl]-4-yl)oxy)ethoxy)ethoxy)ethoxy)ethyl)triphenylphosphonium bromide

Mito-PEG$_4$-HNK was prepared in two steps, by reacting the appropriate PEGylated dibromoalcane with honokiol in the presence of potassium carbonate in DMF. The addition of triphenylphosphine on the bromopegylated honokiol (HNK-PEG$_4$-Br) led to Mito-PEG$_4$-HNK (Supplementary Figure 2).

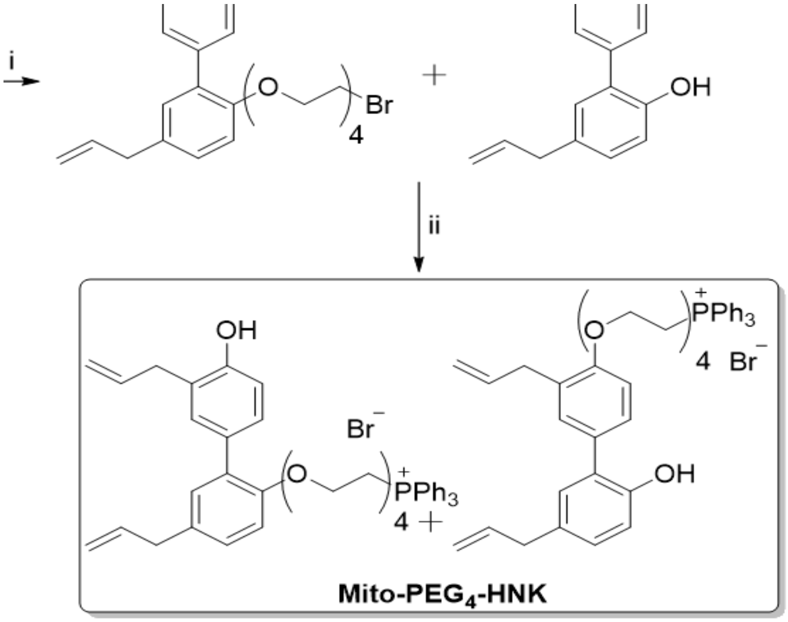

Supplementary Figure 2. Synthesis of Mito-PEG$_4$-HNK. Reagents and conditions: i, 1,11-Dibromo-3,6,9-trioxaundecane, K_2CO_3, DMF, 40°C, 36h, 62%; ii, triphenylphosphine, CH_3CN, reflux, 18h, 31%; iii, 10-Bromodecyltriphenylphosphonium bromide, K_2CO_3, DMF, 60°C, 31%.

To a mixture of honokiol (0.3 g, 1.2 mmol), anhydrous potassium carbonate (0.3 g, 2.4 mmol) in DMF (4 mL) was added 1,11-Dibromo-3,6,9-trioxaundecane (0.36 g, 1.1 mmol). The mixture was stirred at 40 °C for 36h. The residue was taken up into water and extracted with Et_2O. The organic layer was dried over sodium sulfate, and the solvent was removed under reduced pressure. Purification by flash chromatography (Pentane/Et_2O, 1/1) delivered the corresponding PEG-HNK (0.35 g, 62% yield). PEG-HNK was directly used for the next step. A mixture of PEG-HNK (0.35 g, 0.69 mmol) and triphenylphosphine (0.24 g, 0.91 mmol) in acetonitrile (2 mL) was stirred at reflux for 18 hours. The mixture was poured in 100 mL of ether. The precipitate was purified by flash chromatography (CH_2Cl_2 /EtOH 9/1) and led to the corresponding **Mito-PEG$_4$-HNK** (165 mg, 31% yield).

HRMS calculated for **Mito-PEG$_4$-HNK** $C_{44}H_{48}O_5P^+$ $[M]^+$ 687.3234, found, 687.3238.
^{31}P NMR (400.13 MHz, $CDCl_3$) δ 25.39, 25.16. ^{1}H NMR (400.13 MHz, $CDCl_3$), δ ^{1}H NMR (400.13 MHz, $CDCl_3$) δ 7.79-7.67 (9H, m), 7.64-7.56 (6H, m), 7.32 (1H, ddd, J = 2.2, 4.4, 8.0), 7.24 (1H, 2d, J = 2.2, 2.2), 7.17 (1H, 2d, J = 8.0), 7.12-6.94 (2H, m), 6.89-6.80 (1H, m), 6.06-5.89 (2H, m), 5.11-4.95 (4H, m), 4.20-4.01 (2H, m), 3.99-3.80 (5H, m), 3.80-3.61 (3H, m), 3.50-3.40 (2H, m), 3.39-3.28 (5H, m), 3.27-3.15 (4H, m). ^{13}C NMR (75 MHz, $CDCl_3$) δ 155.6, 154.3, 154.0, 151.8, 138.0, 137.8, 137.4, 136.8, 134.6, 134.57, 134.48, 134.4, 134.0, 133.9, 133.8, 132.5, 131.3,

130.9, 130.8, 130.7, 130.1, 130.0, 129.9, 129.8, 129.3, 128.9, 128.4, 128.3, 128.2, 127.5, 127.4, 125.4, 119.3, 119.2, 118.5, 118.4, 116.4, 115.6, 115.2, 115.1, 112.6, 111.6, 71.0, 70.8, 70.5, 70.2, 70.18, 70.10, 69.99, 69.93, 69.66, 69.63, 68.6, 68.0, 63.9, 63.8, 63.7, 63.6, 39.4, 34.5, 25.3 (d, J = 52.1), 25.2 (d, J = 52.8).

Analysis of L-DOPA and Dopamine using LC-MS or LC-MS-SIM

The freeze-dried samples were prepared as follows: To the tubes containing the cells was added 200 µL of ammonium formate buffer (10 mM). Then, the tubes were shaken vigorously (vortex) for 10 s and centrifugated for 7 min × 20,000 g at room temperature. The supernatants (100 µL) were transferred into HPLC vials with conical inserts and analyzed by LC-MS, described as follows:

Mobile phase:
Mobile phase A, 10 mM ammonium formate, pH 3.0, 90% MeCN, 10% water
Mobile phase B, 10 mM ammonium formate, pH 3.0, 50% MeCN, 50% water

Gradient:

Time (min)	Mobile phase A (%)	Mobile phase B (%)
0	100	0
14	20	80
15	100	0
20	100	0
Flow rate: 0.5 mL/min		
Injection volume: 2.0 µL		

Calculated log P values for MTD Analogs

The calculated log P values of the MTD analogs were assessed using a QSAR (quantitative structure–activity relationship) analysis and rational drug design as a measure of molecular hydrophobicity (Supplementary Table 1). This method also uses a consensus model built using the ChemAxon software (San Diego, CA) [4,5].

^{31}P NMR, $CDCl_3$

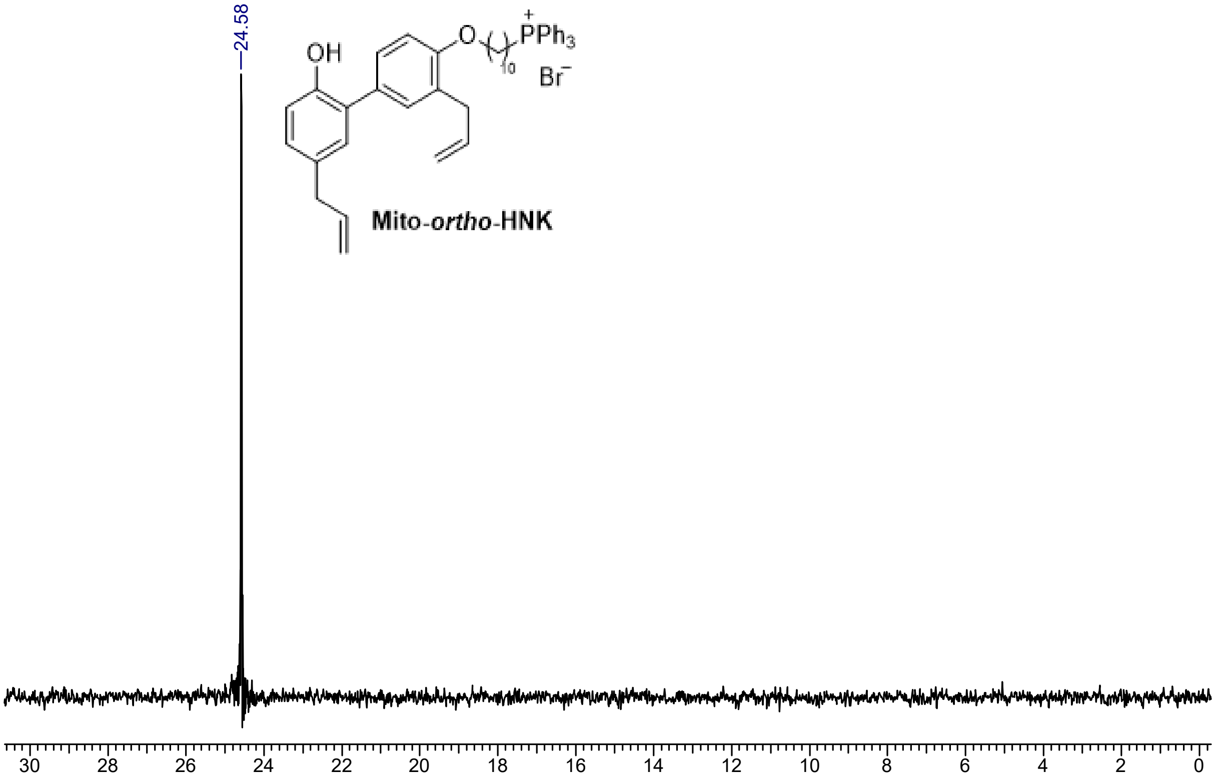

^{1}H NMR, $CDCl_3$

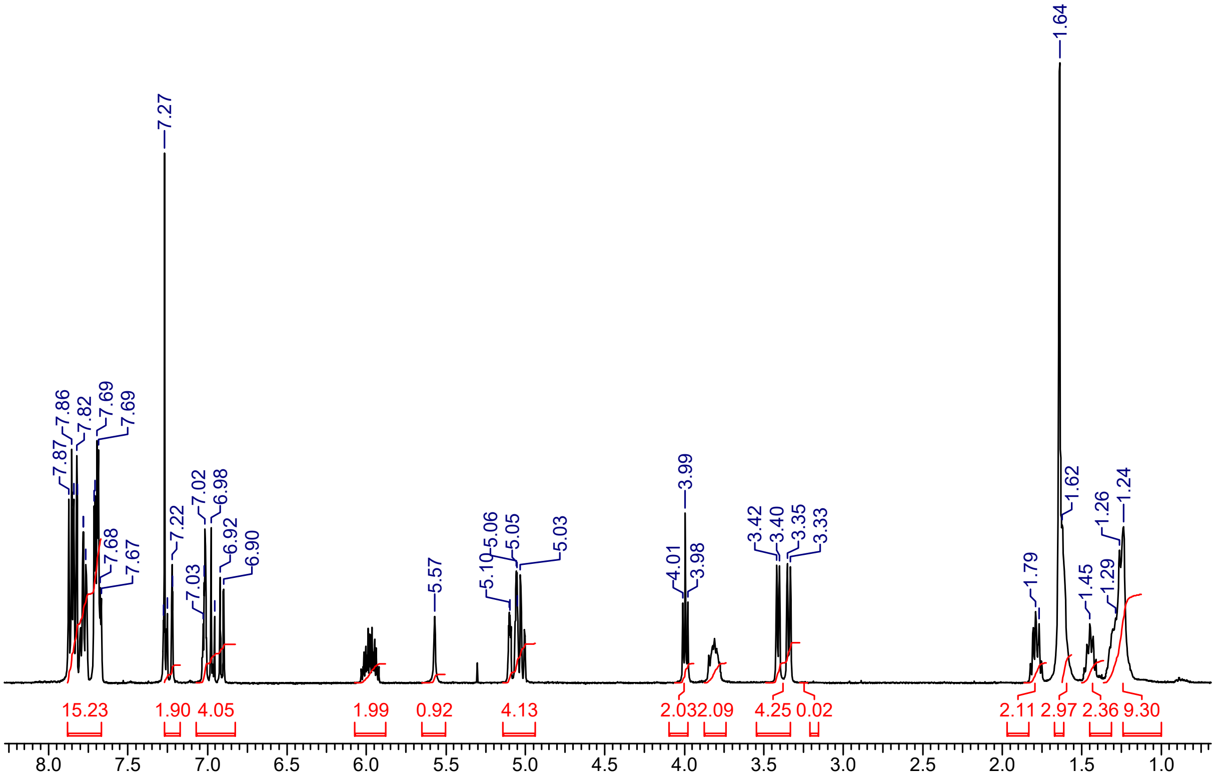

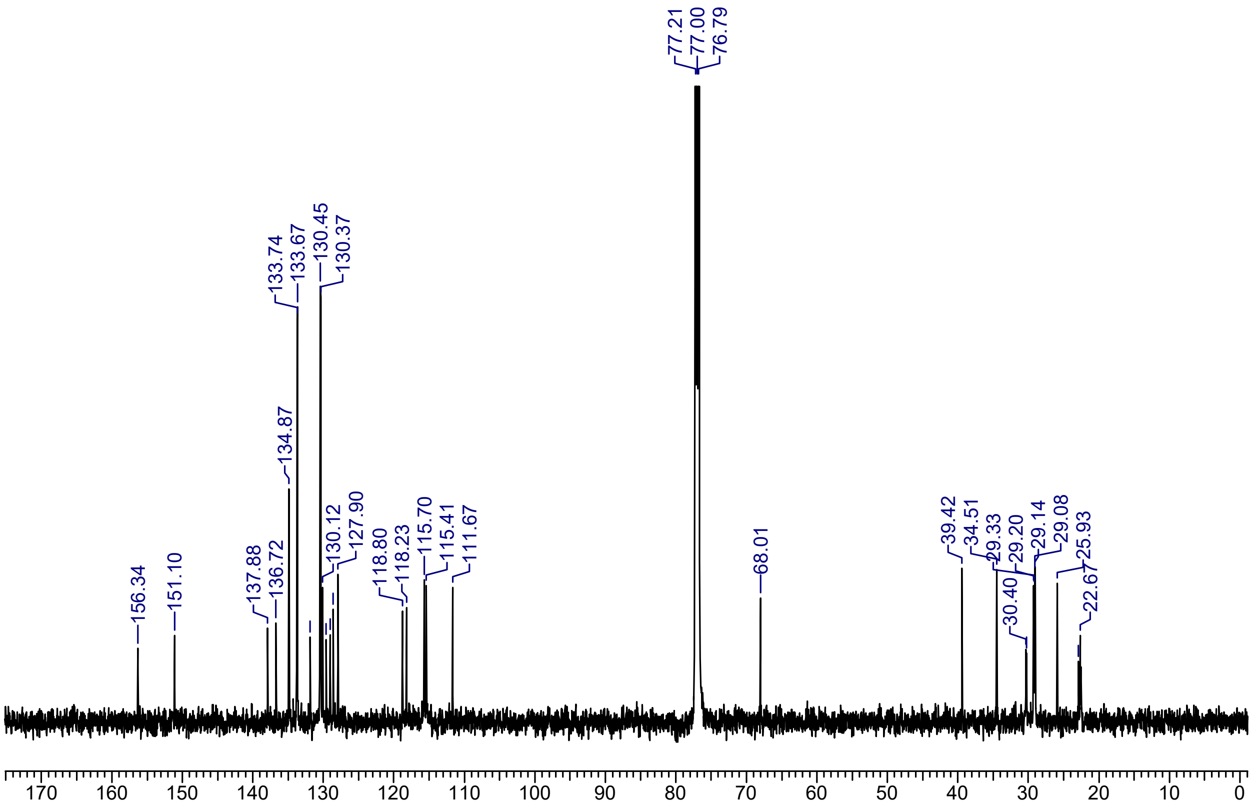

156.34
151.10
137.88
136.72
134.87
133.74
133.67
130.45
130.37
130.12
127.90
118.80
118.23
115.70
115.41
111.67
77.21
77.00
76.79
68.01
39.42
34.51
29.33
29.20
30.40
29.14
29.08
25.93
22.67
170 160 150 140 130 120 110 100 90 80 70 60 50 40 30 20 10 0

COSY

HSQCed

HMBC

NOE

^{31}P NMR (400.13 MHz, $CDCl_3$)

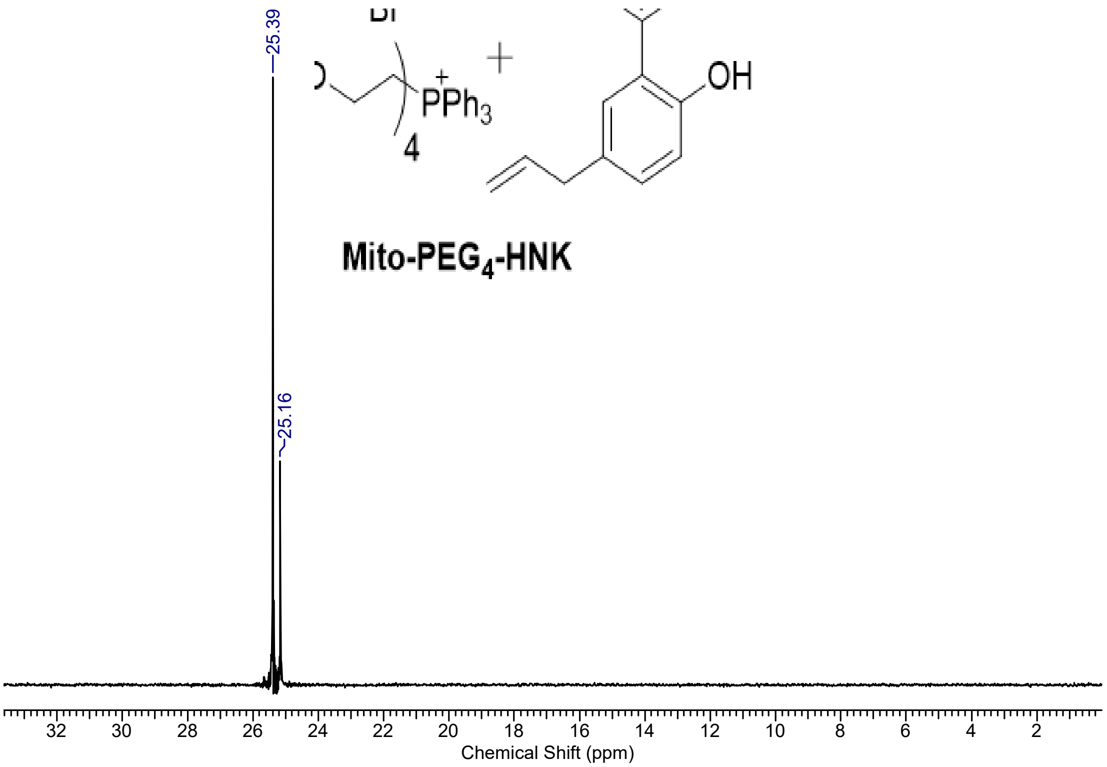

^{1}H NMR (400.13 MHz, $CDCl_3$)

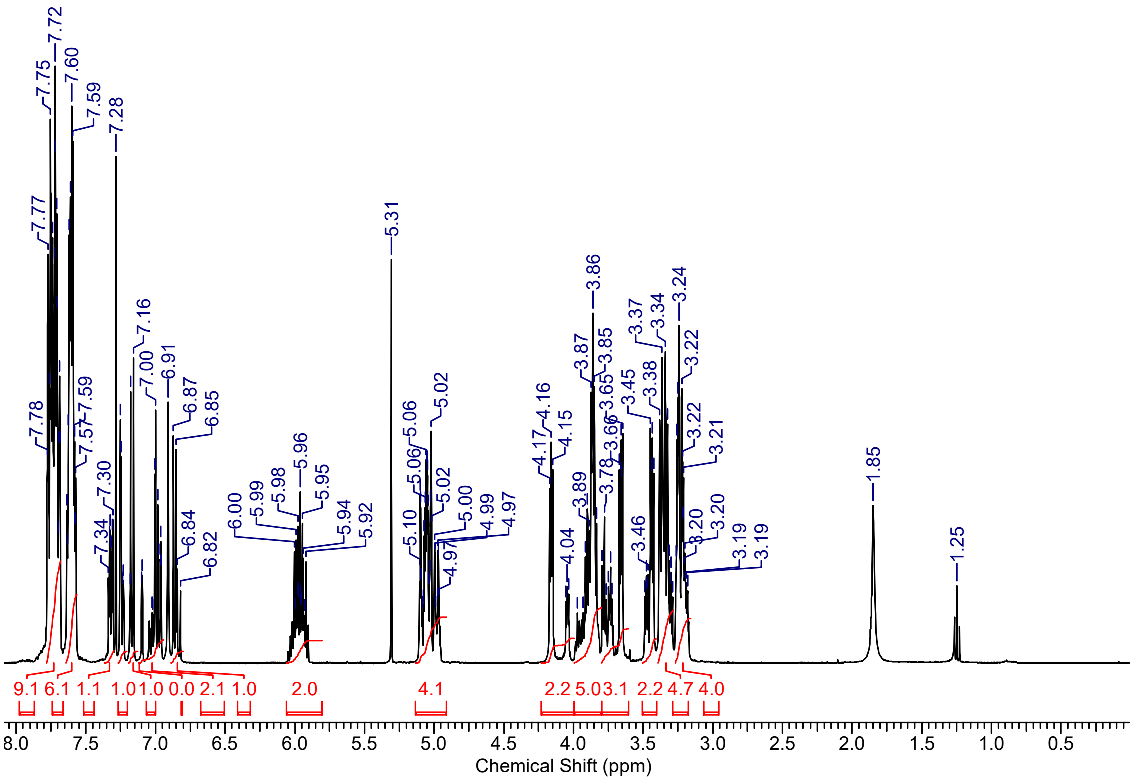

^{13}CAPT (75 MHz, $CDCl_3$)

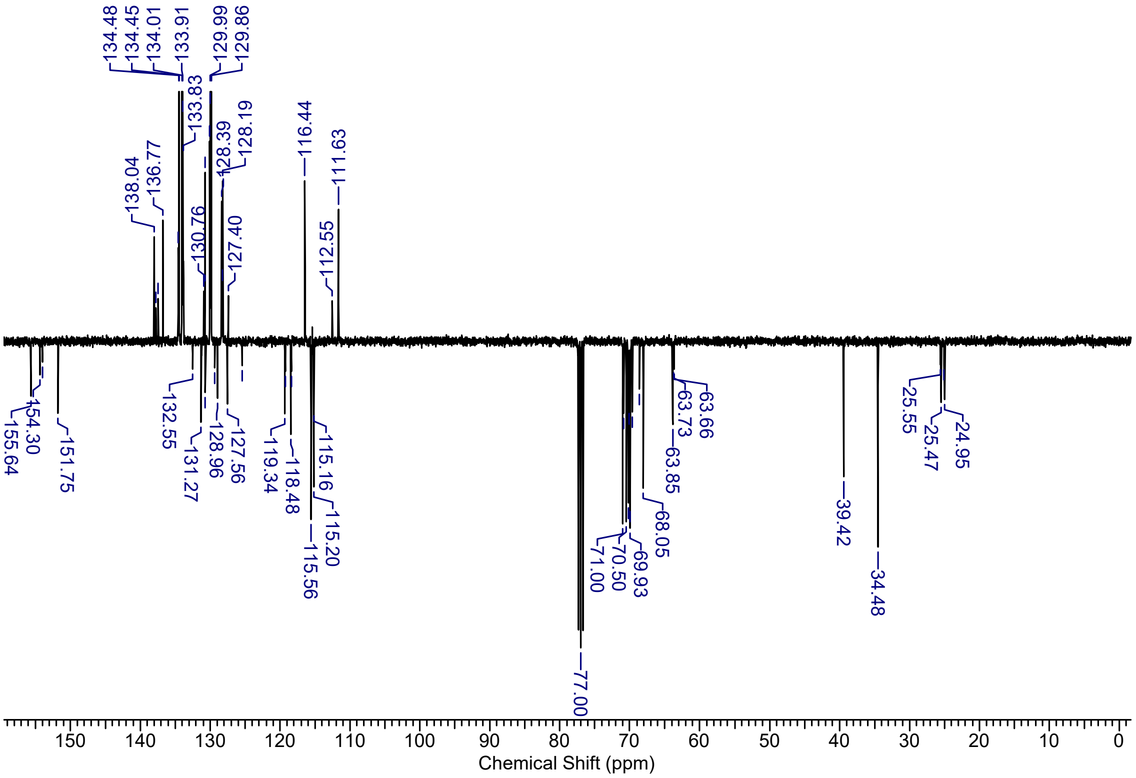

Supplementary Figure 3. NMR data for Mito-*ortho*-HNK and Mito-PEG$_4$-HNK.

Molecules	Structure	logP*
**Atovaquone (ATO)**	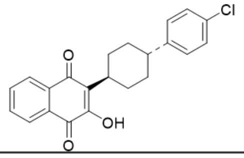	**5.1**[37]
**Honokiol (HNK)**	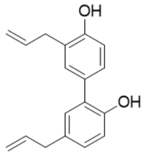	**5.2**
**Lonidamine (LON)**	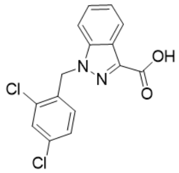	**4.5**
**$Mito_{10}$-Ato**	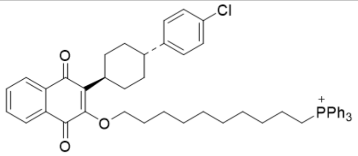	**12.8**[37]
**$Mito_{10}$-HNK**	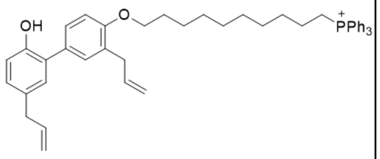	**13.0**
**Mito-LON**	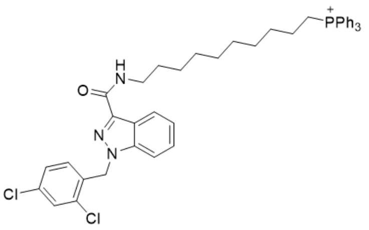	**9.3**
**Mito-PEG_5-ATO**	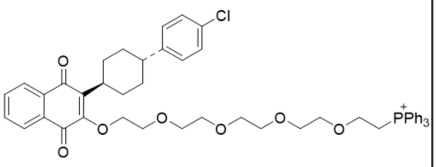	**9.2**[37]
**Mito-PEG_4-HNK**	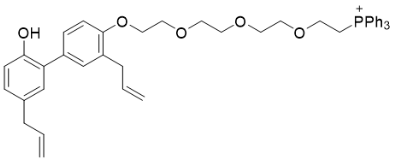	**9.5**
**Mito-PEG_4-APO**	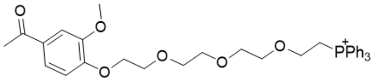	**5.4**

Supplementary Table 1. Calculated values of the octanol/water partition coefficients.

Supplementary References

1 Huang, M. *et al.* Prevention of Tumor Growth and Dissemination by In Situ Vaccination with Mitochondria-Targeted Atovaquone. *Adv Sci (Weinh)* **9**, e2101267 (2022). https://doi.org:10.1002/advs.202101267

2 Cheng, G., Hardy, M., You, M. & Kalyanaraman, B. Combining PEGylated mito-atovaquone with MCT and Krebs cycle redox inhibitors as a potential strategy to abrogate tumor cell proliferation. *Sci Rep* **12**, 5143 (2022). https://doi.org:10.1038/s41598-022-08984-6

3 Pan, J. *et al.* Mitochondria-Targeted Honokiol Confers a Striking Inhibitory Effect on Lung Cancer via Inhibiting Complex I Activity. *iScience* **3**, 192-207 (2018). https://doi.org:10.1016/j.isci.2018.04.013

4 Viswanadhan, V. N., Ghose, A. K., Revankar, G. R. & Robins, R. K. Atomic physicochemical parameters for three dimensional structure directed quantitative structure-activity relationships. 4. Additional parameters for hydrophobic and dispersive interactions and their application for an automated superposition of certain naturally occurring nucleoside antibiotics. *Journal of Chemical Information and Computer Sciences* **29**, 163-172 (1989). https://doi.org:10.1021/ci00063a006

5 Klopman, G., Li, J.-Y., Wang, S. & Dimayuga, M. Computer Automated log P Calculations Based on an Extended Group Contribution Approach. *Journal of Chemical Information and Computer Sciences* **34**, 752-781 (1994). https://doi.org:10.1021/ci00020a009
