# Supplementary material for: Mitigating gut microbial degradation of levodopa and enhancing brain dopamine: Implications in Parkinson’s disease
Source: Commun Biol. 2024 May 30;7:668. doi: 10.1038/s42003-024-06330-2 (PMC11139878; doi:10.1038/s42003-024-06330-2)
Supplement: Supplementary file 3 — Description of Additional Supplementary Files [file 42003_2024_6330_MOESM3_ESM.pdf]

Description of Additional Supplementary Files

File name: Supplementary Data 1

Description: Source data used to generate the main figures.
